# A Rare Lung Malignancy in a Case of Systemic Sclerosis

**DOI:** 10.7759/cureus.65146

**Published:** 2024-07-22

**Authors:** Syed Akram C, Harshavardhini P, Nalini Jayanthi

**Affiliations:** 1 Department of Respiratory Medicine, SRM Medical College Hospital and Research Centre, Chennai, IND

**Keywords:** pulmonary hypertension, usual interstitial pneumonia (uip), progressive pulmonary fibrosis, idiopathic pulmonary fibrosis, adenosquamous carcinoma, interstitial lung disease, systemic sclerosis

## Abstract

Systemic sclerosis (SSc) is one of the chronic autoimmune diseases characterized by the infiltration of excess collagen in various organs, especially the skin. It is found to be associated with a higher prevalence of internal malignancies, particularly lung carcinoma. Herein, we report a case of adenosquamous carcinoma confining within the lung in a patient who had long-standing SSc. She was a 55-year-old female patient presenting with progressive dry cough and breathlessness for six months. She had been a known case of diffuse cutaneous SSc for over a decade, based on 2013 American College of Rheumatology (ACR) criteria. The diagnosis is made based on her findings of bilateral thickening of the fingers on both hands, extending up to the metacarpophalangeal joints. Furthermore, she had telangiectasia at the upper chest wall and neck, multiple pitting scars at the toes, Raynaud's esophageal dilatation, and interstitial lung disease (ILD). She had been treated on Mycophenolate Mofetil 500 mg twice daily and low-dose prednisolone 5 mg once daily for 10 years. The patient's high-resolution computed tomography (HRCT) of the chest revealed a subpleural nodule in the posterior basal segment of the left lower lobe with areas of reticular opacities and interlobular septal thickening on bilateral lung fields six months earlier. The current computed tomography of the lung revealed a new 2.6 x 2.5 cm ill-defined lesion with irregular margins at the left lower lobe. A CT-guided biopsy was done for the lesion, which revealed adenosquamous carcinoma. Immunohistochemistry was consistent with a diagnosis of primary pulmonary adenosquamous carcinoma. The patient did not accept any further investigations and/or treatment. Herein, we present a rare lung malignancy, adenosquamous carcinoma of the lung with an underlying long-term diffuse cutaneous SSc in a nonsmoking female, which highlights the importance of lung cancer screening in individuals with SSc complicated with ILD and supports the fact that there is an increased prevalence of lung cancer among SSc-ILD patients than that of the regular population.

## Introduction

Systemic sclerosis (SSc), also known as scleroderma, is a rare connective tissue disorder with a complex and unknown pathogenesis. It can be categorized into two main types: localized scleroderma and SSc. Localized scleroderma includes subtypes such as morphea, linear scleroderma, and scleroderma en coup de sabre, while SSc is divided into limited SSc (formerly CREST syndrome) and diffuse SSc based on clinical and serological criteria. Limited SSc is characterized by calcinosis, Raynaud phenomenon, esophageal dysmotility, sclerodactyly, and telangiectasia, whereas diffuse SSc involves more widespread skin thickening [1].

Limited cutaneous SSc affects skin distal to the elbows and knees or the face without trunk involvement. Diffuse cutaneous SSc involves skin thickening in areas proximal to the elbows, knees, face, and trunk. Both types of SSc can affect various organs and are associated with positive autoantibodies, including antinuclear antibodies in over 90% of cases and specific autoantibodies in up to 70%. SSc can affect the skin, gastrointestinal tract, lungs, kidneys, skeletal muscle, and pericardium, with overlapping manifestations with other rheumatological or immunological diseases.

The association between SSc and cancer is complex and suggests that SSc may act as a paraneoplastic syndrome in some patients, similar to dermatomyositis [2,3]. This relationship is influenced by multiple mechanisms. While immunosuppressants used to treat SSc can potentially lead to cancer [4-6], cytotoxic anti-cancer therapies have been linked to the development of scleroderma-like features such as the Raynaud phenomenon, digital ischemia, and fibrosis [7,8]. Shared mechanisms and pathways might be involved in both fibrogenesis and oncogenesis, with recent evidence suggesting that autoimmunity in SSc may be triggered by antigen mutations in tumor cells, potentially enhancing anti-tumor defenses. The use of immune checkpoint inhibitors in cancer treatment, which can trigger autoimmune responses, further highlights the role of immunity in cancer development and progression. These findings underscore the intricate bilateral relationships between SSc and cancer. Adenosquamous carcinoma (ASC) is a rare type of lung cancer, comprising 0.4%-4% of all lung carcinomas. It is known for its more aggressive behavior and poorer prognosis [9,10].

## Case presentation

A 55-year-old nonsmoking female and homemaker with a decade-long history of diffuse cutaneous SSc complicated by interstitial lung disease (ILD) and cor pulmonale over the past two years presented with increasing dyspnea grade III-IV on the modified Medical Research Council dyspnea scale and dry cough over a year. She had a history of bluish discoloration and numbness of the extremities on cold exposure and complaints of retrosternal burning sensation over 10 years. Furthermore, she had worsening respiratory systems with physiological and radiological evidence of progressive pulmonary fibrosis over the course. She had been receiving immunosuppressive therapy with Mycophenolate Mofetil 500 mg twice daily and a low dose of prednisolone 5 mg once daily for SSc, T. Nintedanib 150 mg BD as an anti-fibrotic treatment for progressive fibrosing ILD (ILD) and T. Ambrisentan 5 mg along with T. Tadalafil 20 mg once daily for pulmonary hypertension. She gets an injection of denosumab every six months to manage osteoporosis. Furthermore, she requires domiciliary oxygen therapy at a rate of 2 L per minute via nasal prongs for 15-18 hours daily.

**Figure 1:**
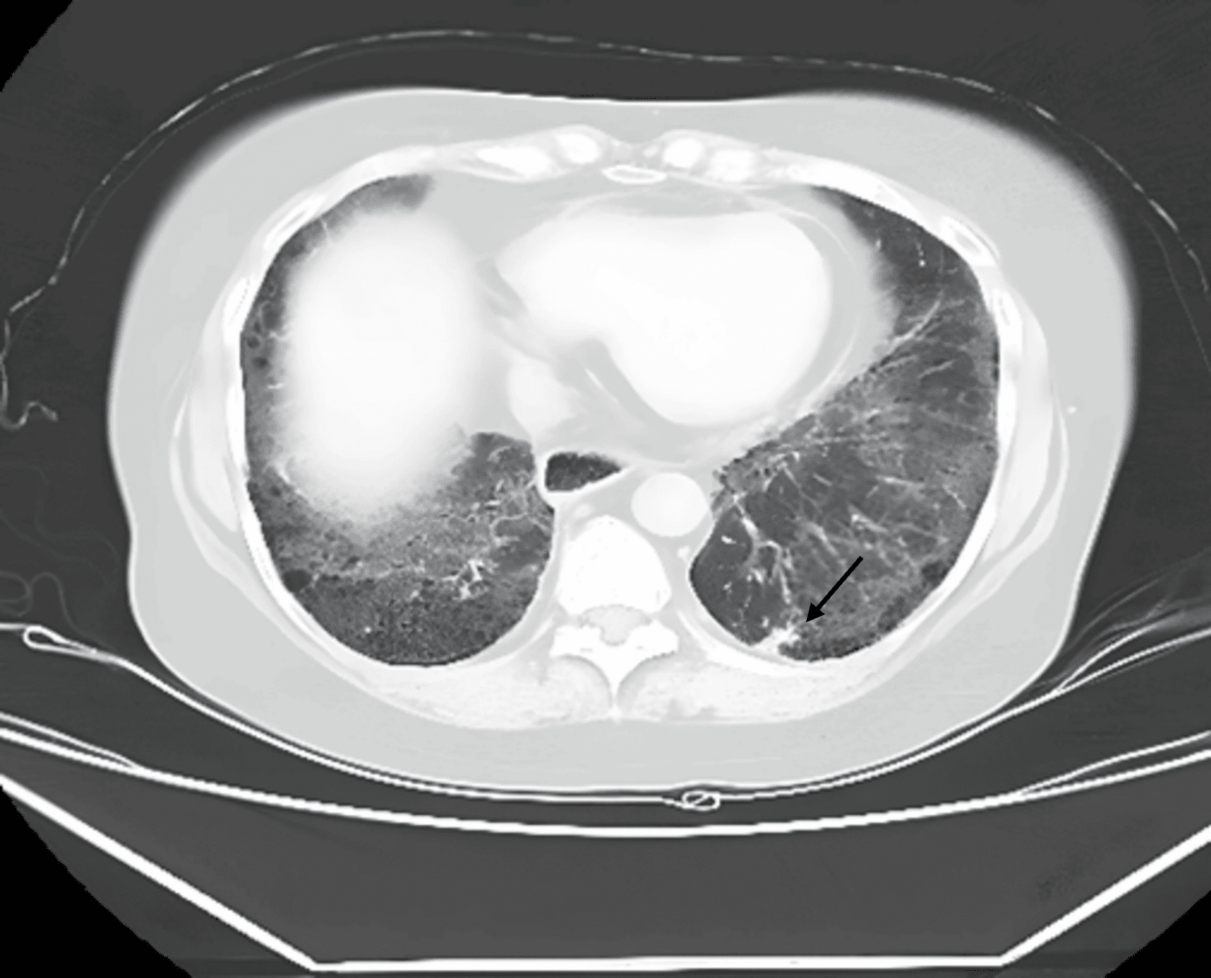
The CT of the chest shows interstitial lung disease (probable UIP)—bibasilar subpleural reticulations, interlobar septal thickening, and mild GGOs with a left lower lobe subpleural nodule (1.1 x 0.4 cm) in the posterobasal segment UIP: usual interstitial pneumonia; GGO: ground-glass opacity

**Figure 2:**
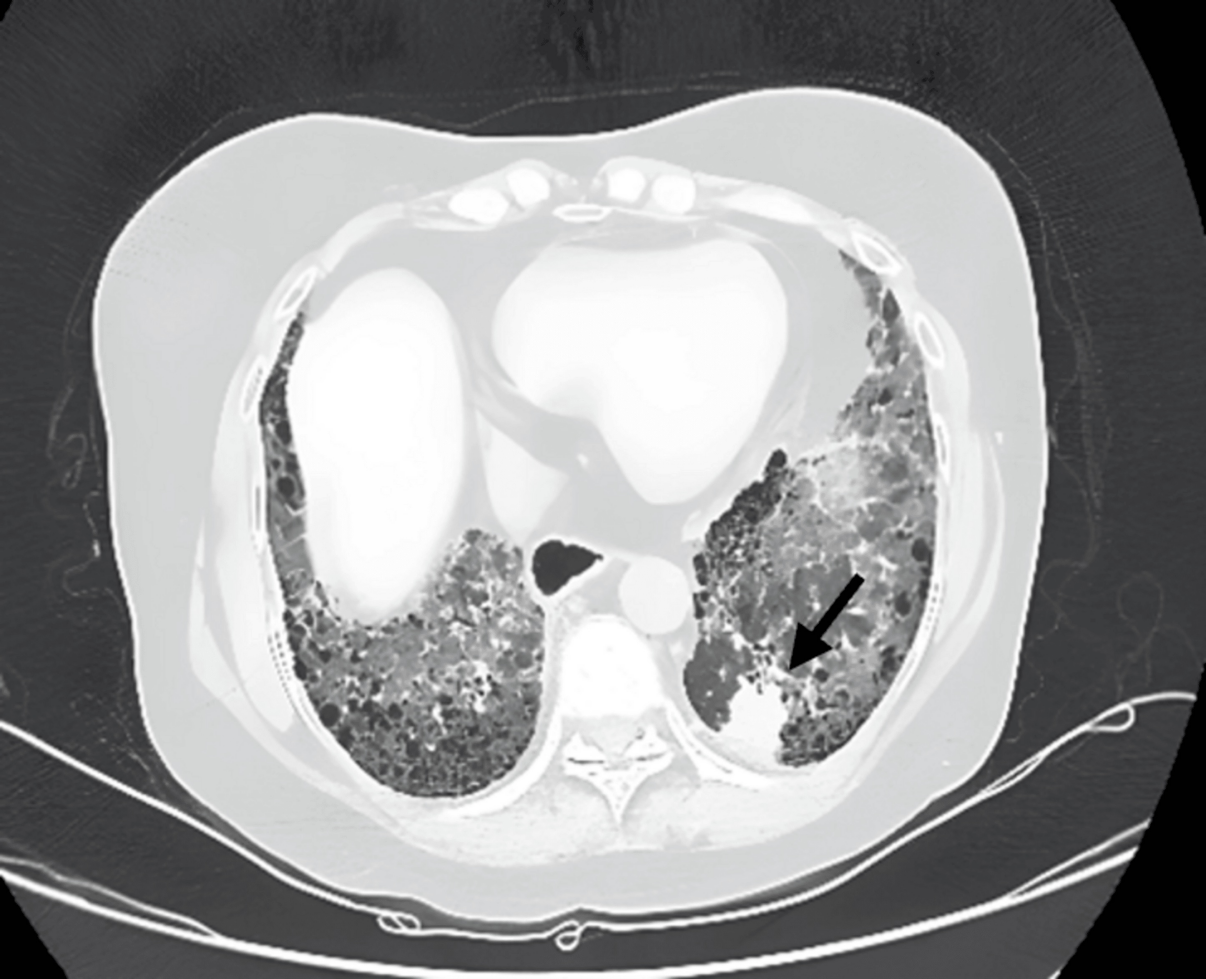
A follow-up CT of the chest shows a radiological progression of the interstitial lung disease (UIP), bilateral lower lobe subpleural honeycombing, reticular opacities, interlobular septal thickening, and an ill-defined, heterogeneously enhancing lesion in the left lower lobe posterobasal segment, measuring 2.5 x 2.6 cm

The clinical examination revealed bilateral thickening of the fingers on both hands extending up to the metacarpophalangeal joints, telangiectasia over the chest and neck, and erythematous papules on her face, forearms, and legs, along with digital ulcers in bilateral toes consistent with the existing diagnosis. Additionally, a respiratory assessment revealed bilateral basal Velcro crackles. The initial investigations showed normal leukocyte count, liver, and renal function. The arterial blood gas analysis indicated type I respiratory failure with a PaO2 of 52 mmHg on room air. The 2D echocardiography showed pulmonary hypertension with a right atrium and ventricular dilation accompanied by severe tricuspid regurgitation, while a chest X-ray revealed bilateral lower zone reticular opacities with cardiomegaly. On the prior visit six months ago, the patient's high-resolution computed tomography (HRCT) of the chest revealed a 1.1 x 0.4 cm subpleural nodule with fibrosis in the posterior basal segment of the left lower lobe (Figure 1). Notably, in the current visit, the patient's HRCT of the chest revealed a 2.6 x 2.5 cm ill-defined lesion with irregular margins and adjacent pleural thickening at the same site of the nodule in the posterior basal segment of the left lower lobe (Figure 2). This finding raised significant concern for malignancy and subsequent scrutiny of the lesion.

**Figure 3:**
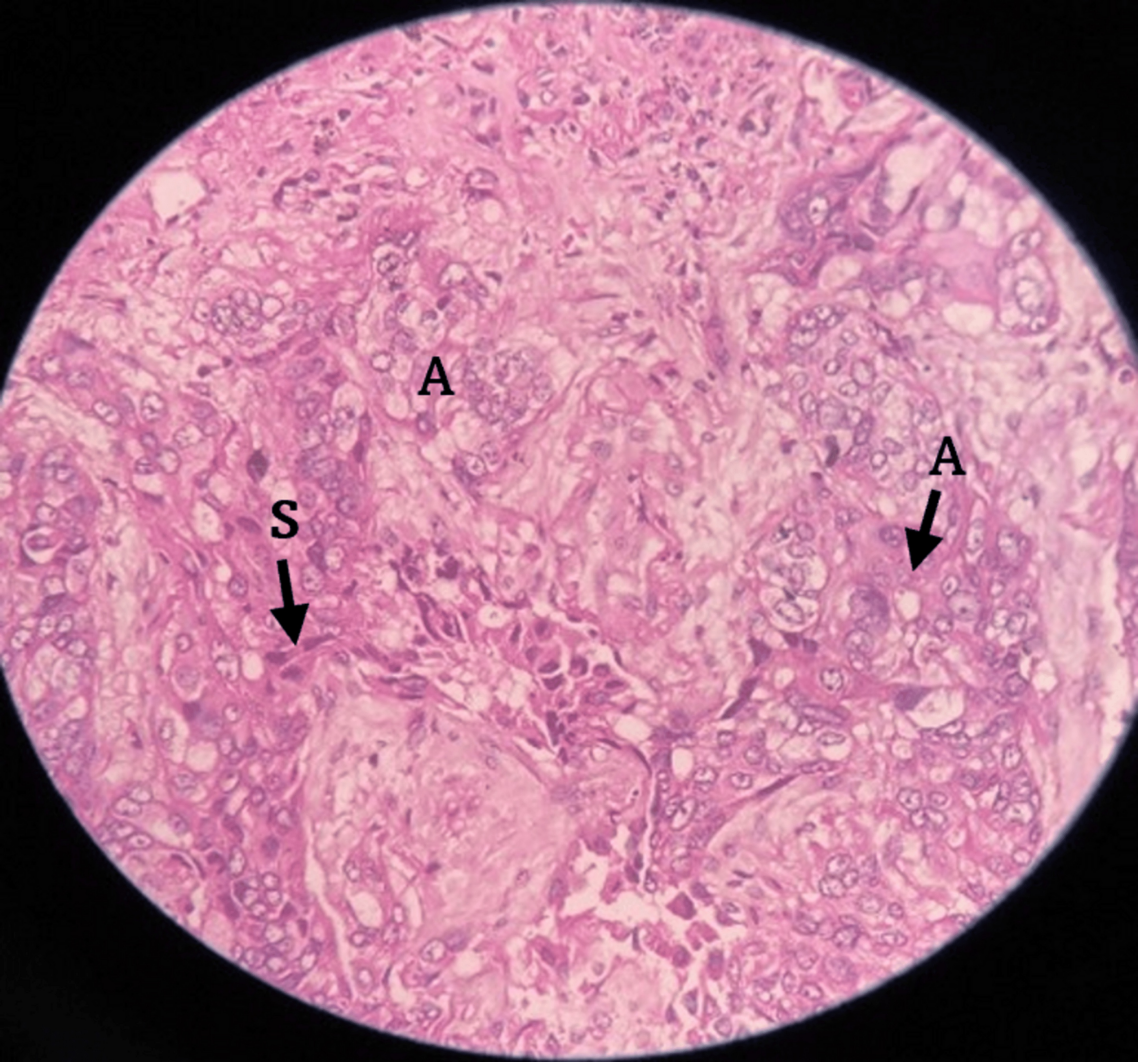
A 40× H&E image of a lung biopsy shows strands and islands of squamous(S) and adenomatous (A) components of the adenosquamous carcinoma in a fibrous background H&E: hematoxylin and eosin

A CT-guided lung biopsy of the lesion confirmed the presence of a poorly differentiated adenosquamous carcinoma, as evidenced by the histopathological examination (Figure 3). Immunohistochemistry revealed positivity for thyroid transcription factor-1 (TTF-1), aspartic proteinase A (NAPSIN A), P40, P63, cytokeratin 7 (CK7), and focal positivity for carcinoembryonic antigen (CEA), consistent with a diagnosis of primary pulmonary adenosquamous carcinoma.

## Discussion

The involvement of the lungs in SSc is an important cause of mortality, either directly in the form of ILD or pulmonary hypertension (PH) or indirectly in the form of malignancy, aspiration, infection, or drug toxicity [11]. Several theories have been suggested regarding the development of lung cancer in SSc. These include chronic inflammation, immunosuppressive treatments with cytotoxic effects, impaired immune surveillance and carcinogen clearance, encounters with prevalent environmental stimuli, and genetic inclination toward both carcinogenesis and autoimmune dysregulation [12-16].

Lung cancer incidence in SSc patients has been consistently reported for a considerable duration, and the link between ILD and lung malignancy is firmly established in the literature. The most common histological types include adenocarcinoma and squamous cell carcinoma. However, rare occurrences have been documented, such as the case reported by Kanaji et al., who described a 51-year-old nonsmoking woman with SSc-associated interstitial pneumonia diagnosed with small-cell lung cancer [17], and Sugiura et al. reported a case of solitary organizing pneumonia mimicking lung adenocarcinoma in SSc [18]. In this particular case, a nonsmoking female diagnosed with diffuse cutaneous SSc with Raynaud phenomenon complicated by PH that developed secondary to the ILD, which led to the cor pulmonale, presented with worsening shortness of breath and cough and was diagnosed as a rare case of primary pulmonary adenosquamous carcinoma. Adenosquamous carcinoma typically exhibits clinical features of both adenocarcinoma and squamous cell carcinoma, often associated with a dismal prognosis [19]. This case underscores the diagnostic challenge for patients with underlying autoimmune conditions and ILD, where respiratory symptoms may mask the onset of malignant processes. It is recommended to have an HRCT examination in patients with SSc to look for evidence of ILD and be vigilant for any lung malignancy in the early stages of the disease.

## Conclusions

SSc-ILD is linked to a heightened risk of lung cancer. The above case underscores the significant association between SSc and lung carcinoma. Identifying the shared pathogenic mechanisms driving both conditions and recognizing the risk factors for lung carcinoma in SSc strengthens an active collaboration between rheumatologists and pulmonologists. Early diagnosis is crucial in both SSc and lung cancer, emphasizing the necessity for integrated management by pulmonologists, rheumatologists, and pathologists to capitalize on treatment opportunities and optimize patient outcomes, particularly in terms of survival.
